# Development and alpha-testing of a patient decision aid for patients with chronic myeloid leukemia regarding dose reduction

**DOI:** 10.1186/s12911-024-02806-7

**Published:** 2024-12-20

**Authors:** D. N. Lokhorst, M. F. Djodikromo, R. P. M. G. Hermens, N. M. A. Blijlevens, C. L. Bekker

**Affiliations:** 1 Department of Hematology, Radboud University Medical Center, Nijmegen, The Netherlands; 2 IQ Health, Radboud University Medical Center, Nijmegen, The Netherlands; 3 Department of Pharmacy, Radboud University Medical Center, Geert Grooteplein Zuid 10, 6525 GA Nijmegen, The Netherlands

**Keywords:** Dose reduction, Tyrosine kinase inhibitors, Chronic myeloid leukemia, Clinical decision support, Shared decision making

## Abstract

**Background:**

Dose reduction of tyrosine kinase inhibitors (TKIs) is an option for some chronic myeloid leukemia (CML) patients to minimize side effects while maintaining efficacy. Shared decision-making (SDM) and patient decision aids (PDAs) are advocated to make informed choices such as reducing the dose of TKIs. This paper describes the development and alpha-testing of a PDA for patients with CML receiving TKI dose reduction.

**Methods:**

The PDA was iteratively developed following IPDAS guidelines. First, a needs assessment with semi-structured interviews was conducted to understand the needs and preferences of patients and healthcare providers. Second, through feedback cycles with the project team and steering group the scope, content, and format were defined. Third, three rounds of alpha-testing were performed via individual “think aloud” sessions with patients (round 1) and healthcare providers (round 2) to qualitatively assess the comprehensibility, acceptability, and desirability of the PDA. Round 3 included quantitative evaluation via an acceptability and usability questionnaire. Qualitative data were categorized, and quantitative data were descriptively analyzed.

**Results:**

The majority valued the development of the PDA during the needs assessment (*n* = 30). The PDA included disease and treatment information, information about dose reduction, knowledge questions, and a value clarification section. During alpha-testing, the PDA was considered clear, balanced, and helpful for decision-making. A total of 76% of the patients (*n* = 17) and 100% of the healthcare providers (*n* = 9) recommended it with overall mean scores of 7.4 and 7.8, respectively. The above average usability score was 68.1.

**Conclusion:**

A well-accepted online PDA for chronic phase CML patients to consider TKI dose reduction was developed.

**Supplementary Information:**

The online version contains supplementary material available at 10.1186/s12911-024-02806-7.

## Background

Treatment with tyrosine kinase inhibitors (TKIs) has significantly improved survival outcomes in patients with chronic myeloid leukemia (CML). Patients receiving adequate treatment approach life expectancies comparable to those of the general population [1]. The treatment strategy for CML involves many considerations, ranging from selecting the initial treatment to adjusting doses or switching medications if side effects become unbearable [2]. Each treatment option has its own risk-benefit profile, which can be evaluated differently by patients and healthcare providers (HCPs) [3]. The reduction of TKI dosage for patients in stable remission has been explored as a potential approach to minimize side effects while maintaining treatment effectiveness [4–6]. Cheng et al. recently reviewed clinical trials and real-world studies focusing on the dose optimization of imatinib, dasatinib, and nilotinib [7]. Additionally, dose adjustments for bosutinib and ponatinib have been explored by Castagnetti et al. [8], Latagliata et al. [9], and Jabbour et al. [10]. Importantly, reducing the TKI dose has been shown to significantly improve the quality of life and mental health of patients with CML. As such, dose reduction could be an option for some patients, but with this choice benefits (e.g., fewer side effects) and risks (e.g., disease progression) are associated that need careful consideration of both patients and healthcare providers. To ensure that the preferences and values of patients are taken into account, it is crucial to involve patients in the decision-making process [11]. To make such preference-sensitive decisions, shared decision-making (SDM) is advocated [12].

SDM involves patients and healthcare providers in the decision-making process, considering the best available evidence and the patient’s values and preferences [12]. SDM is recognized as an important component of patient-centered care, especially in the context of CML [11, 13]. SDM involves four steps: (1) the healthcare provider communicates the possibility for a treatment decision, emphasizing the relevance of the patient’s input; (2) evaluation of the treatment options, including pros and cons; (3) discussion of patient’s preferences with the healthcare provider assisting in the deliberation; and (4) addressing the patient’s decision-making desire and either making or postponing the decision, followed by a discussion of the next steps [12].

SDM could facilitate discussions between patients and healthcare providers, which could focus on the potential positive and negative aspects of dose reduction, the risk of disease progression, and patient’s tolerance of treatment-related side effects. In fact, patients with hematologic malignancies tend to overestimate the risks and benefits of treatment [3]. Therefore, straightforward information to enhance patients’ understanding of risks and consequences is needed. Patient decision aids (PDAs) can help facilitate the SDM process by providing evidence-based information on all aspects and consequences of treatment choices [14, 15]. PDAs are tools designed to support patients by making their decisions explicit, providing information on available options and their (dis)advantages, and helping to clarify the coherence between decisions and individual values and preferences [14]. The demonstrated benefits include improved patient knowledge, reduced decisional conflict, and increased involvement in decision-making [14]. To date, no PDA is available for patients with CML who are considering TKI dose reduction. Therefore, this study aimed to develop and alpha test a PDA for patients with CML to consider TKI dose reduction.

## Methods

### Study design and setting

The development and evaluation of a PDA was part of the RODEO study, which focuses on the development and evaluation of a patient guided dose reduction strategy for TKI in patients with CML (EudraCT: 2021-006581-20, 10.1186/s12885-023-10697-6) [16].

**Fig. 1:**
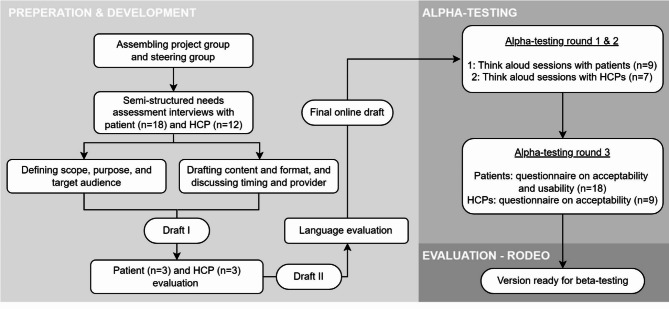
Development process of the PDA. Beta-testing and analysis of the PDA is part of the RODEO study [16]. PDA: Patient Decision Aid, HCPs: Healthcare providers

The International Patient Decision Aids Standards (IPDAS) criteria were used to guide the development process [17]. Additionally, recommendations by Coulter et al. and the guidelines for PDAs from the Dutch Patient Federation were consulted for further guidance [18, 19]. The development process is summarized in Fig. 1 and consists of three key elements in iterative cycles: understanding users, developing and refining the prototype, and observing user interactions, as described by Vaisson et al. [20]. Development was performed by an interdisciplinary project group, consisting of a hematologist, a pharmacist, a patient advocate, and three researchers in pharmacy, implementation science and epidemiology. Next, an interdisciplinary steering group, which served as an advisory board throughout the development process, was formed. This group consisted of seven members, including two patient representatives affiliated with a patient association, two hematologists, one pharmacist, and two nurse specialists.

### Needs assessment

To comprehensively understand the needs and preferences of both patients and healthcare providers regarding the content and format of the PDA, a qualitative study was conducted among patients with CML and healthcare providers (hematologists, pharmacists, and hematology nurses) until data saturation was achieved. After providing written consent, semi-structured interviews were conducted in which participants were questioned about their general view of TKI dose reduction, the advantages and disadvantages of TKI dose reduction, the perceived roles of healthcare providers and patients in the decision process, and their preferences and needs for a PDA. The interview guide can be found in Appendix A. Since the scope of this study is the development of the PDA, insights from the interviews into the necessity of a PDA and the desired information were extracted. A detailed evaluation of the full interview results will be presented elsewhere.

### Scope, purpose, and target audience

The PDA was developed in a process of co-creation and feedback between the project and the steering group. During development, the findings from the needs assessment interviews were considered and evaluated. The project group initially defined the scope, purpose, and target audience of the PDA, and this was discussed with the steering group during an online meeting.

### PDA content and format

Based on the identified needs and suggestions during the needs assessment interviews, the project and steering group discussed the content, format, and timing of provision and provider of the PDA. Thereafter, a draft was created and evaluated by three patients and three healthcare providers. These participants received the PDA draft and were asked to fill out a questionnaire regarding the content (Appendix B). Input was used to improve the PDA. Furthermore, the PDA was evaluated by an organization with experience in adjusting medical texts for patients with low literacy (www.stichtingmakkelijklezen.nl, *easy-to-read foundation*). Thereafter, a final online version was developed and ready for alpha-testing.

### Alpha-testing

The main objective of alpha-testing was to assess the comprehensibility (degree to which the content of the PDA is understandable), acceptability (degree to which the PDA adds value to the consultation), feasibility (degree to which the PDA would fit into clinical practice), and desirability (degree to which the PDA was presented in a visually appealing way). The participants in alpha-testing consisted of patients with CML and healthcare providers who were not involved in the previous development process. Three separate rounds were conducted. In all rounds, the demographic characteristics of the participants were recorded.

### Alpha-testing rounds 1 and 2 – think aloud sessions

Round 1 - Adult patients with CML using a TKI were eligible for inclusion for alpha-testing, both patients who already had reduced their dose and naïve patients. Patients were recruited from five hospitals via their healthcare providers. After providing written consent, patients received the online PDA, and an online interview was held. The participants were asked to provide verbal feedback via the “think aloud” method, by saying out loud thoughts and impressions regarding the PDA. Patients were subsequently asked about the content, layout, comprehensibility, usability, and acceptability guided by a topic list (Appendix C).

Round 2 - Healthcare providers involved in CML care (hematologists, residents, and nurses) from eight hospitals were invited to participate via email. After providing written consent, a “think aloud” session followed by a short interview was held, with a focus on the practicality and desirability of PDA in clinical pathways (Appendix C).

### Alpha-testing round 3 – acceptability and usability

In a third alpha-testing round, the final draft of the PDA was quantitatively evaluated among CML patients and healthcare providers. Patients were recruited via a CML care platform (www.CMyLife.nl), and healthcare providers were recruited via email invitations. The participants were asked to complete an online questionnaire about the acceptability and comprehensibility of the PDA (Appendix D). In addition, patients were asked to rate the usability of the PDA via the system usability scale (SUS) (Appendix E). For acceptability, the acceptability scale was used to assess how well information was presented for different topics and overall impressions of the PDA. The questionnaire included questions regarding the length, amount of information, balance of the PDA, and participants’ opinions about the content of the PDA [21]. The SUS is a 10-item questionnaire with a 5-point Likert scale (ranging from “Strongly disagree” to “Strongly agree”) to assess the subjective usability of a product [22]. The total SUS score can vary between 0 and 100 where a score above 68 is higher than the average SUS score [23].

### Data analysis

Semi-structured interviews and think-aloud sessions were audio-recorded and transcribed verbatim. Two independent researchers coded and categorized the data. Each comment raised by the participants was documented and discussed with the project group who decided which suggestions had to be incorporated to revise the PDA. Qualitative and quantitative data were analyzed with ATLAS.ti (version 8) and RStudio 4.1.3., respectively. Descriptive statistics were used to describe the quantitative data. Continuous data were expressed as the means with standard deviations (SDs) or medians with ranges. Categorical data were expressed as proportions.

## Results

### Needs assessment

The interviews were held with eighteen patients and twelve healthcare providers (six hematologists, four hematology nurse specialists, and two pharmacists). Most patients and healthcare providers expressed support for TKI dose reduction for various reasons such as reduced side effects, lower medication costs and fewer side effects. Most patients hoped or expected that dose reduction would lead to more freedom and ability in daily life. The primary concern about TKI dose reduction for both patients and healthcare providers is centered around the potential recurrence of the disease through the loss of effectiveness of TKI treatment. To make a well-informed decision, information about personal possibilities for dose reduction and potential consequences and risks was found crucial by patients. Participants emphasized that the decision-making process should always be conducted as collaboration between the patient and healthcare provider, specifically the hematologist as appointed by patients. Participants highlighted the patient’s sense of trust with the healthcare provider as absolute must for effective SDM. Both patients and healthcare providers valued the development of a user-friendly PDA, particularly to prepare and inform patients for consultation. In addition, patients would use it as a summary and reference for what is discussed during the consultation. Some healthcare providers highlighted that the use of a PDA and SDM is only applicable for decisions that are not made for medical necessity. Patients and healthcare providers would like to see the risks, benefits, and consequences of dose reduction in the PDA. In addition, real-world or clinical trial data on the success rate of dose reduction were deemed essential by both patients and healthcare providers. Patients preferred the information to be as simple as possible, whereas healthcare providers would like to see scientific references. Also, patients would value the inclusion of positive and negative patient experience stories regarding dose reduction.

### Scope, purpose, and target audience

The scope, purpose, and target audience were defined by the project group in accordance with the steering group. The scope of the PDA was to improve informed decision making by patients to reduce TKI dosage, serving a two-fold purpose: supporting patients with CML in deciding on TKI dose reduction and preparing them for a SDM consultation with a healthcare provider on this topic. The identified target audience for the PDA included adult patients with chronic-phase CML treated with TKIs (imatinib, dasatinib, bosutinib, nilotinib, ponatinib) with a sustained major molecular response (MMR) (BCR-ABL1 levels ≤ 0.1%) for ≥ 6 months. The project and steering group agreed that the PDA should be provided by the CML healthcare provider, particularly the hematologist or hematology nurse. The tool was intended for use by patients at home before their consultation with a hematologist or hematology nurse.

### PDA content and format

During the development process, it was decided by the project group, in accordance with the steering group and guided by the needs assessment interviews, to create an online PDA with the option to have a printed version if requested by patients. The final online draft consisted of six steps: (1) information about TKI dose reduction; (2) differences and similarities between dose reduction and no dose reduction; (3) knowledge check; (4) questions about personal considerations that play a role in the decision for dose reduction; (5) evaluation and decision making; and (6) a summary sheet of the completed PDA with options to directly print, save or email the results. Table 1 presents a detailed description of each step. Additionally, links directing patients to external websites for further information were included, and each step included a dedicated section for personal notes. The PDA content was written according to the B1 level of the Common European Framework of Reference for Languages (CEFRL).

**Table 1:** Description of steps included in the PDA

Step		Content
1	Introduction	A. Background information: Information about eligibility of TKI dose reduction, the purpose of dose reduction and a link to CML care platform CMyLife for detailed information about CML and its treatment. B. Dose reduction: More detailed information is provided. Information is structured to answer the following questions: What does dose reduction entail? Do I have to choose dose reduction? C. Results: Information is structured to answer the following questions: What happens to my CML? What is the effect of dose reduction on side effects? Is it possible to experience side effects from dose reduction? D. Possibilities: Information is structured to answer the following questions: Is it still possible to switch from TKI in the future? Is a treatment free remission attempt still possible?
2	Differences and similarities	An overview of differences or similarities between dose reduction or not is given for the following topics: extra blood sampling, consequences for CML, effects on side effects, possibility to switch from TKI in the future, and possibility for a treatment free remission attempt in the future.
3	Knowledge check	Four statements about dose reduction with real-time feedback on the answers given are presented. Topics include effect on side effects, blood sampling, consequences of increased BCR-ABL1 levels, and consequences for a future treatment free remission attempt.
4	Evaluation and decision making	With a slider, patients are asked to what extent they agree/disagree with statements about side effects, extra blood sampling, importance of medication quantity, concerns about potential increased CML activity, and concerns regarding a potential treatment free remission attempt.
5	Expectations and concerns	With open and closed questions patients are asked about their concerns about dose reduction, what side effects they experience currently, if they understand enough about the (dis)advantages, if they know what pros and cons are most important to them, and they are asked to specify information they want from their healthcare provider. Finally, patients are asked what treatment option they prefer.
6	Summary	A summary sheet consisting of the patient’s personal considerations, preferences, and questions for the healthcare provider. The sheet can be printed, saved as PDF and emailed.

### Alpha-testing

Alpha-testing was performed with 27 patients and 16 healthcare providers in three rounds. The participant characteristics are presented in Table 2.

**Table 2:** Sociodemographic characteristics of the participants in alpha-testing

	Alpha-testing	
**Patients**	Round 1 (*n* = 9)	Round 3 (*n* = 18)
Age (mean, SD)	55.1 (12.4)	63.8 (11.3)
Sex, male	5 (55.6%)	14 (77.8%)
Educational level		
High Middle Low	5 (55.6%) 4 (44.4%) -	6 (33.3%) 10 (55.6%) 2 (11.1%)
TKI treatment		
Imatinib Dasatinib Bosutinib Nilotinib	3 (33.3%) 4 (44.4%) - 2 (22.2%)	9 (50%)* 4 (22.2%) 1 (5.6%) 3 (16.7%)
Patients receiving a lower dose	1 (11.1%)	15 (83.3%)
Type of hospital undergoing treatment		
Academic medical center Top clinical General hospital	2 (22.2%) 7 (77.8%) -	5 (27.8%) 11 (61.1%) 2 (11.1%)
**Healthcare providers**	Round 2 (*n* = 7)	Round 3 (*n* = 9)
Sex, female	100%	N/A
Profession		
Hematologist Physician-specialist (internal medicine) in training Hematology nurse practitioner	7 (100%) - -	5 (55.6%) 2 (22.2%) 2 (22.2%)
Hospital		
Academic medical center General hospital	4 (57.1%) 3 (42.9%)	1 (11.1%) 8 (88.9%)
Years of experience		
0–5 > 5	N/A	4 (44.4%) 5 (55.6%)

* one missing value for the imatinib dosage

### Alpha-testing rounds 1 and 2 – think aloud sessions

Twelve healthcare providers were contacted, and seven agreed to participate, resulting in a response rate of 58.3%. In general, patients (*n* = 9) and healthcare providers (*n* = 7) considered the PDA to be relevant and user-friendly. Both groups found the PDA informative and would recommend it to patients considering TKI dose reduction. In addition, they were positive about the layout, and the information was perceived as comprehensible. Although healthcare providers missed referencing to evidence-based studies supporting the content, patients preferred plain, straightforward information; therefore, it was decided by the project group to not include this information. Indeed, most patients appreciated the amount of information they received. Healthcare providers found the PDA valuable for patients to prepare for consultation and expected additional benefits for patient empowerment. Both patients and healthcare providers believed that the personal summary sheet supported SDM and that it could help patients make value- and preference-based choices.

### Alpha-testing round 3 – acceptability and usability questionnaire

Seventeen out of 52 approached patients (32.7%), and nine out of twenty approached healthcare providers (45%) completed the questionnaire. The PDA scored positive on various acceptability items (see Table 3). Overall, the PDA was found to be helpful in decision making by both patients (64.7%) and healthcare providers (88.9%) and contains enough information to make a decision, as agreed upon by 82.4% of the patients and 77.8% of the healthcare providers. Additionally, the clarity of each section was rated well (Appendix F). All healthcare providers and the majority of the patients (76.5%) would recommend the PDA to others and scored the PDA with mean scores of 7.4 and 7.8, respectively. The SUS yielded a mean score of 68.1 (SD 17). See Appendix G for the SUS per scale item. Based on these evaluations, the PDA was updated and ready for beta-testing by removing any double negative statements and improving the online navigation.

**Table 3:** Acceptability of the PDA scored by both patients and healthcare providers

Acceptability item	Patients (*n* = 17)	HCP (*n* = 9)
Time spent in minutes, mean (SD)	9.7 (7.8)	10.6 (3.9)
Length		
Too long Just right Too short	2 (11.8%) 14 (82.4%) 1 (5.9%)	1 (11.1%) 8 (88.9%) -
Amount of information		
Too little Just right Too much	1 (5.9%) 15 (88.2%) 1 (5.9%)	- 7 (77.8%) 2 (22.2%)
Information balanced?		
Yes No, favoring dose reduction No, favoring no dose reduction	14 (82.4%) 3 (17.6%) -	5 (55.6%) 2 (22.2%) 2 (22.2%)
General comprehensibility PDA		
Good Average Poor	15 (88.2%) 1 (5.9%) 1 (5.9%)	8 (88.9%) 1 (11.1%) -
Are potential disadvantages of dose reduction comprehensible?		
Good Average Poor I miss this information	14 (82.4%) - 2 (11.8%) 1 (5.9%)	8 (88.9%) 1 (11.1%) - -
Is the information appropriate for patients?		
Good Average Poor	15 (88.2%) - 2 (11.8%)	7 (77.8%) 2 (22.2%) -
How is the navigation through the PDA?		
Easy Neutral Difficult	14 (82.4%) 1 (5.9%) 2 (11.8%)	9 (100%) - -
The PDA consists confusing items (yes)	3 (17.6%)	3 (33.3%)
The personal value clarification step…		
Made the choice easier Made the choice harder Does not influence my choice	4 (23.5%) - 13 (76.5%)	8 (88.9%) - 1 (11.1%)
The PDA is helpful in decision-making (yes)	11 (64.7%)	8 (88.9%)
The PDA has enough information to make a decision (yes)	16 (82.4%)	7 (77.8%)
Score on a scale 1–10 (mean, SD)	7.4 (1.6)	7.8 (0.7)
Recommend the use of the PDA (yes)	13 (76.5%)	9 (100%)

## Discussion

In this study, a PDA for patients with chronic phase CML to discuss TKI dose reduction was developed and evaluated. The PDA was based on the needs of patients and healthcare providers, and was considered clear, balanced, and helpful for decision-making by both patients and healthcare providers during alpha-testing. The amount of information, length, presentation, and clarity of information received positive feedback. The PDA received a high recommendation score of 76.5% by patients and 100% by healthcare providers. The usability of the PDA score was just above average of 68.1 according to Sauro and Lewis [24].

Many patients would value the inclusion of positive and negative patient experience stories regarding dose reduction. Nonetheless, it was decided to not include these in the final PDA, which was supported by literature, as this has many practical challenges and might elicit persuasion [25]. As the body of information concerning TKI dose reduction continues to expand, the PDA necessitates regular updates to ensure its relevance and accuracy.

PDAs can aid in SDM, especially in preference-sensitive decisions. As identified in the needs assessment interviews, some healthcare providers may only see the value of SDM and PDAs in these cases and not in situations where decisions are made for medical necessity. However, it is important to recognize that SDM should ideally be integrated into all consultations, as patients always retain the option to decline a specific treatment option, regardless of medical necessity. Nonetheless, using a PDA can be particularly advantageous when careful consideration of patients’ preferences is needed to explore different treatment options.

Evidence suggests that optimal trust between patients with cancer and their oncologist can positively affect patients’ treatment experience and outcomes [26]. Indeed, in the needs assessment interviews, many patients emphasized the vital importance of the bond of trust with their healthcare provider, often nurtured through a prolonged therapeutic relationship, for open communication and effective SDM. While the PDA cannot replace this, it can empower patients to engage in informed discussions with their healthcare provider, thus fostering more effective discussions. Therefore, PDAs serve as an educational, informative, and supportive tool in these interactions. Additionally, it is important to emphasize that PDAs are not a replacement for direct consultations. The healthcare provider should always review the information presented in the PDA with the patient to ensure full understanding and proper informed consent. This collaborative approach ensures that patients are equipped to make well-informed decisions about their care.

The main strengths of this study are the variety of participant groups involved during the systematic development process and the use of a user-centered methodology with pilot testing, as recommended by the International Patient Decision Aid Society [17, 18, 20]. Both patients and healthcare providers could express their needs and first impressions of the PDA in semi-structured interviews and “think aloud” sessions, respectively, as well as providing feedback on its acceptability. The development was carefully executed by a multidisciplinary project group with extensive experience in various areas, such as the clinical setting and implementation research, and monitored by an interdisciplinary steering group. Another notable strength of this study is the diversity observed within the study population, encompassing patients of various ages, educational backgrounds, and receiving treatment across different types of hospitals. This ensures that the PDA will be acceptable for the entire Dutch-speaking CML patient population. Also, the healthcare provider study population included various professions, years of experience and activities in both academic and peripheral hospitals. The efforts to write the PDA content for patients with low health literacy hold significant value, as they directly address the need for greater attention to health literacy in the field of PDAs [27].

The PDA was developed in Dutch. This poses a constraint for patients with limited or no proficiency in the Dutch language, potentially hindering their ability to fully engage with the decision-making process and comprehend the provided materials. Therefore, this limitation could reduce the accessibility and applicability of the PDA for an international population. In this study, cultural differences were not specifically addressed, even though certain information may be interpreted or valued differently across cultures. Due to the rarity of CML, it was not feasible to incorporate cultural diversity into the participant selection process. The scoring method might have influenced the SUS. Here, the standard SUS was used, although Lewis et al. advocated for the use of the positive version of the SUS [23]. This is recommended as this version reduces the likelihood of response or scoring errors, particularly during surveys or unmoderated remote usability studies, as conducted in this research. Future research should keep this in mind. When considering the findings of this study, it is imperative to acknowledge the potential for selection bias in the recruitment methodology. Using a patient platform naturally attracts patients who are more actively engaged in managing their health. Indeed, several patients in alpha-testing round one mentioned involvement in other clinical studies as well as their generally high involvement in CML management. Although the increased engagement of this group may offer more informed critical evaluations of the PDA, it also presents a limitation. Specifically, the lack of viewpoints from patients who might have less knowledge or involvement could distort the results and lead to an incomplete representation of the varied experiences and opinions concerning the PDA. This could be seen as an opportunity to explore accessibility and usability in a broader patient population. This will be performed in the next step, beta-testing, to assess the PDA in a real-world setting. For this purpose, the PDA underwent one last update to improve usability by eliminating double negative statements and improving the integration of all the elements. Beta-testing is part of the currently ongoing RODEO study with expected results in 2026 [16].

## Conclusion

A PDA for patients with chronic-phase CML who consider TKI dose reduction was successfully developed and well accepted by both patients and healthcare providers. Upon clinical implementation, the PDA will undergo beta-testing in the currently ongoing RODEO study.

## Electronic supplementary material

Below is the link to the electronic supplementary material.

Supplementary Material 1

## Data Availability

The quantitative data supporting the findings of this study are available within the article and its supplementary materials. The qualitative data of alpha-testing are available form the corresponding author on reasonable request. The qualitative data of the needs assessment are available after publication of these results.

## Abbreviations

HCP: Healthcare Provider
TKI: Tyrosine Kinase Inhibitor
CML: Chronic Myeloid Leukemia
SDM: Shared Decision-Making
PDA: Patient Decision Aid
IPDAS: International Patient Decision Aids Standards
SUS: System Usability Scale
MMR: Major Molecular Response
CEFRL: Common European Framework of Reference for Languages

## References

[1] Bower H, Björkholm M, Dickman PW, Höglund M, Lambert PC, Andersson TM-L. Life expectancy of patients with chronic myeloid leukemia approaches the life expectancy of the General Population. J Clin Oncol. 2016;34(24):2851–7. 27325849 10.1200/JCO.2015.66.2866

[2] Senapati J, Sasaki K, Issa GC, Lipton JH, Radich JP, Jabbour E, Kantarjian HM. Management of chronic myeloid leukemia in 2023 – common ground and common sense. Blood Cancer J. 2023;13(1):58. 37088793 10.1038/s41408-023-00823-9 PMC10123066

[3] El-Jawahri A, Nelson-Lowe M, VanDusen H, Traeger L, Abel GA, Greer JA, Fathi A, Steensma DP, LeBlanc TW, Li Z, et al. Patient-clinician discordance in perceptions of treatment risks and benefits in older patients with Acute myeloid leukemia. Oncologist. 2019;24(2):247–54. 30139841 10.1634/theoncologist.2018-0317 PMC6369944

[4] Clark RE, Polydoros F, Apperley JF, Milojkovic D, Pocock C, Smith G, Byrne JL, de Lavallade H, O’Brien SG, Coffey T, et al. De-escalation of tyrosine kinase inhibitor dose in patients with chronic myeloid leukaemia with stable major molecular response (DESTINY): an interim analysis of a non-randomised, phase 2 trial. Lancet Haematol. 2017;4(7):e310–6. 28566209 10.1016/S2352-3026(17)30066-2

[5] Claudiani S, Apperley JF, Szydlo R, Khan A, Nesr G, Hayden C, Dominy AJI, Foskett K, Foroni P. TKI dose reduction can effectively maintain major molecular remission in patients with chronic myeloid leukaemia. Br J Haematol. 2021;193(2):346–55. 33368155 10.1111/bjh.17286

[6] Martín Roldán A, Sánchez Suárez MDM, Alarcón-Payer C, Jiménez Morales A, Puerta Puerta JM. A real-world evidence-based study of long-term tyrosine kinase inhibitors dose reduction or discontinuation in patients with chronic myeloid leukaemia. Pharmaceutics. 2023;15(5):1363. 37242605 10.3390/pharmaceutics15051363 PMC10222180

[7] Cheng F, Li Q, Cui Z, Hong M, Li W, Zhang Y. Dose optimization strategy of the tyrosine kinase inhibitors imatinib, dasatinib, and nilotinib for chronic myeloid leukemia: from clinical trials to real-life settings. Front Oncol. 2023;13:1146108. 37091188 10.3389/fonc.2023.1146108 PMC10113500

[8] Castagnetti F, Bocchia M, Abruzzese E, Capodanno I, Bonifacio M, Rege Cambrin G, Crugnola M, Binotto G, Elena C, Lucchesi A, et al. P698: Bosutinib dose optimization in the second-line treatment of elderly cml patients: extended 3-year follow-up and final results of the best study. HemaSphere. 2022;6:593–4.

[9] Latagliata R, Attolico I, Trawinska MM, Capodanno I, Annunziata M, Elena C, Luciano L, Crugnola M, Bergamaschi M, Bonifacio M, et al. Bosutinib in the real-life treatment of chronic myeloid leukemia patients aged > 65 years resistant/intolerant to previous tyrosine-kinase inhibitors. Hematol Oncol. 2021;39(3):401–8. 33617659 10.1002/hon.2851

[10] Jabbour E, Apperley J, Cortes J, Rea D, Deininger M, Abruzzese E, Chuah C, DeAngelo DJ, Hochhaus A, Lipton JH, et al. Dose modification dynamics of ponatinib in patients with chronic-phase chronic myeloid leukemia (CP-CML) from the PACE and OPTIC trials. Leukemia. 2024;38(3):475–81. 38287132 10.1038/s41375-024-02159-0 PMC10912029

[11] Clements J, Fleischman A, Lerner V, Ruiz C. The importance of developing open communication and a professional, long-term relationship between patients with chronic myeloid leukemia and their oncologist. Future Oncol. 2023;19(17):1197–208. 37218534 10.2217/fon-2022-1267

[12] Stiggelbout AM, Pieterse AH, De Haes JC. Shared decision making: concepts, evidence, and practice. Patient Educ Couns. 2015;98(10):1172–9. 26215573 10.1016/j.pec.2015.06.022

[13] Geerts PAF, van der Weijden T, Moser A, Bos GMJ. The Perception of Shared decision-making in Hematology by patients and Physicians seems satisfactory, but important steps are still ahead of us. Hemasphere. 2020;4(4):417. 10.1097/HS9.0000000000000417 PMC7430231 32885141

[14] Stacey D, Légaré F, Lewis K, Barry MJ, Bennett CL, Eden KB, Holmes-Rovner M, Llewellyn-Thomas H, Lyddiatt A, Thomson R, Trevena L. Decision aids for people facing health treatment or screening decisions. Cochrane Database Syst Rev. 2017;4(4):Cd001431. 28402085 10.1002/14651858.CD001431.pub5 PMC6478132

[15] O’Connor AM, Légaré F, Stacey D. Risk communication in practice: the contribution of decision aids. BMJ. 2003;327(7417):736. 14512487 10.1136/bmj.327.7417.736 PMC200814

[16] Djodikromo MF, Hermens RP, Bemt B, Smit Y, Govers TM, Bekker CL, Blijlevens NM. Patient-guided dose reduction of tyrosine kinase inhibitors in chronic myeloid leukaemia (RODEO study): study protocol for a prospective, multicentre, single-arm trial. BMC Cancer. 2023;23(1):231. 36899295 10.1186/s12885-023-10697-6 PMC10007754

[17] Resources. [http://ipdas.ohri.ca/resources.html]

[18] Coulter A, Stilwell D, Kryworuchko J, Mullen PD, Ng CJ, van der Weijden T. A systematic development process for patient decision aids. BMC Med Inf Decis Mak. 2013;13:S2. 10.1186/1472-6947-13-S2-S2 PMC4044159 24625093

[19] Hoe maak ik een. keuzehulp bij een richtlijn? [https://www.patientenfederatie.nl/downloads/brochures/493-hoe-maak-ik-een-keuzehulp-bij-een-richtlijn/file]

[20] Vaisson G, Provencher T, Dugas M, Trottier M-È, Chipenda Dansokho S, Colquhoun H, Fagerlin A, Giguere AMC, Hakim H, Haslett L, et al. User involvement in the design and development of patient decision aids and other Personal Health tools: a systematic review. Med Decis Making. 2021;41(3):261–74. 33655791 10.1177/0272989X20984134

[21] O’Connor AMC. A.: User Manual - Acceptability [document on the Internet]. In. Ottawa: Ottawa Hospital Research Institute 1996 [modified 2002].

[22] Brooke J. SUS: A quick and dirty usability scale. In: Usability evaluation in industry. Edited by Patrick W. Jordan BT, Ian Lyall McClelland, Bernard Weerdmeester. London; 1996: 189–194.

[23] Lewis JR. The System Usability Scale: past, Present, and Future. Int J Hum Comput Interact. 2018;34:577–90.

[24] Jeff Sauro JRL. Quantifying the User Experience Practical Statistics for User Research (2nd ed.). In.: Morgan Kaufmann; 2016: 198–209.

[25] Shaffer VA, Brodney S, Gavaruzzi T, Zisman-Ilani Y, Munro S, Smith SK, Thomas E, Valentine KD, Bekker HL. Do personal stories make patient decision Aids more effective? An update from the International patient decision Aids standards. Med Decis Making. 2021;41(7):897–906. 34027739 10.1177/0272989X211011100

[26] Hillen MA, Koning CC, Wilmink JW, Klinkenbijl JH, Eddes EH, Kallimanis-King BL, de Haes JC, Smets EM. Assessing cancer patients’ trust in their oncologist: development and validation of the Trust in Oncologist Scale (TiOS). Support Care Cancer. 2012;20(8):1787–95. 21947560 10.1007/s00520-011-1276-8 PMC3390706

[27] Muscat DM, Smith J, Mac O, Cadet T, Giguere A, Housten AJ, Langford AT, Smith SK, Durand M-A, McCaffery K. Addressing health literacy in patient decision aids: an update from the International patient decision Aid standards. Med Decis Making. 2021;41(7):848–69. 34053361 10.1177/0272989X211011101 PMC8815094

