# Supplementary material for: Development and alpha-testing of a patient decision aid for patients with chronic myeloid leukemia regarding dose reduction
Source: BMC Med Inform Decis Mak. 2024 Dec 20;24:398. doi: 10.1186/s12911-024-02806-7 (PMC11660603; doi:10.1186/s12911-024-02806-7)
Supplement: Supplementary file 1 — Supplementary Material 1 [file 12911_2024_2806_MOESM1_ESM.docx]

# Appendices

## A: Needs assessment semi-structured interview guides

*Interview guide patient*

1. General view (attitude)
2. What do you know about dose reduction of anticancer medication?
3. How do you feel about reducing the dose of your anticancer medication?
4. When do you think dose reduction might be possible?
5. Perceived risks and benefits
6. What do you think would happen if your anticancer medication were reduced?
7. What do you think potential benefits could be with dose reduction?
8. What do you think potential risks could be with dose reduction?
9. What is a reason for you to (not) reduce/continue?
   1. What do you expect with (fewer side effects/changed disease status/reverting dose)?
10. Which reason(s) is most important to you?
11. To what extent do you think it is possible for you to reduce/how likely do you consider it?
12. Needs and preferences
13. What do you need to make this decision?
14. What do you expect from your healthcare provider when making this decision?
15. Who has the decision-making authority regarding dose reduction (patient/doctor)? Why?
16. How would you like to be informed about possible dose reduction?
17. Need for supporting decision aids
18. What do you think of this?
19. What experience do you have with using a decision aid?
20. Would you use a decision aid if it were available?
    1. What are reasons for you to use a decision aid?
    2. What are reasons for you not to use a decision aid?
21. What do you expect from the decision aid?
22. How should the decision aid look?
    1. Format: online/paper/other..?
    2. What information should definitely be included in the decision aid?
23. By whom should the decision aid be offered?

*Interview guide healthcare provider*

1. General view (attitude)
2. What do you know about dose reduction of TKIs?
3. How do you feel about dose reduction of TKIs? To what extent do you apply this to your patients?
4. When do you think dose reduction might be possible? For example, disease timeline, patient characteristics?
5. Perceived risks and benefits
6. What do you think potential benefits could be with dose reduction?
7. What do you think potential risks could be with dose reduction?
   1. What is needed to mitigate these risks?
8. What is a reason for you to (not) reduce the dose?
   1. Which reason weighs most heavily for you?
9. Preferences
10. What do you need to apply/facilitate dose reduction with TKIs? a. For example, evidence on effectiveness, policy adjustments, etc.
11. What is the role of the patient in the decision-making process?
12. What is your role in the decision-making process?
13. Who has the decision-making authority regarding dose reduction (patient/doctor/pharmacist/nurse)? Why? How do you envision that?
14. According to you, which other parties are involved, and what roles do they play?
15. Need for supporting decision aids
16. What do you think of this?
17. What experience do you have with using a decision aid?
18. Would you use a decision aid for dose reduction if it were available? What are reasons for you to use a decision aid? What are reasons for you not to use a decision aid?
19. At what stage of treatment would you use it?
20. How would you use it? For example, during consultations, as assistance with patient questions.
21. Do you think there should be a separate decision aid for healthcare providers and patients? Why (not)?
22. What do you expect from the decision aid?
23. What information should definitely be included in the decision aid(s)? What are the pros/cons of dose reduction? Would you like to see images/videos in the decision tree? If yes, which ones?
24. How should the information be delivered? (paper/online/?)

## B: Questionnaire to evaluate the content of the PDA prior to alpha-testing

1. What is your general opinion about the content of the decision aid?
2. Which parts of the decision aid do you find useful?
3. Which parts of the decision aid do you find superfluous?
4. What information are you missing from the decision aid?
5. Which parts of the decision aid do you find understandable?
6. Which parts of the decision aid do you find unclear? How can we improve these pieces?

## C: Interview guide think aloud sessions

Would you please review the decision aid again and tell me your thoughts on each section? I would like to know what you think of the information, what questions come to mind as you read it, if you find the text clearly written, and your opinion on the layout. How is it to use this tool?

Questions during think-aloud: These questions are intended as 'nudges' to encourage the interviewee to share their thoughts.

Step 1A: What do you think of the introduction? We see that this step is divided into three screens... what are your thoughts on that?

Step 1B: What do you think of this page? What questions does this information raise for you? What do you think of how this information is presented?

Step 1C: What do you think of the information displayed here? What questions does it raise for you? What do you think of the amount of information? There is a table which you can expand by clicking on 'view table' → what do you think of the table?

Step 2: What do you think of this representation? What do you think is missing in the comparison between the two choices?

Step 3: What do you think of the questions? Both the correct and incorrect answers contain explanations. What seems better to you, to leave it like this or to provide explanations only for the incorrect answer?

Step 4: What do you think of the considerations? Are there other considerations that come to mind for you? If yes, which ones?

Step 5: What do you think of the questions asked? Which questions do you think are missing? What do you think of the questions under the heading 'my choice'?

Now I have more general questions: You may have already answered some questions, but I will repeat them to make sure we have covered everything.

- Comprehensibility of content
  - What do you think of the information provided? → Can you explain that? Can you tell me what you find particularly good/bad?
  - Which parts do you find unnecessary? → How can we change this?
  - Which parts should we keep as they are? → What do you like about these parts?
  - What information do you still miss? → What needs to be added in your opinion?
  - What questions does the decision aid still raise for you? → What else can we add to the decision aid?
- Layout
  - What do you think about offering the decision aid online? →
  - How do you find the layout? → what could be improved? → How would this change help, do you think?
  - What do you think of the readability of the text? → What can we do to make the decision aid more readable? → Did you have to enlarge/reduce the text?
  - Do you have any further comments on how the decision aid looks?
- Usability
  - To what extent do you think this decision aid helps in making a choice? → In what way do you think it helps? → if the answer is does not help: can you explain that further? → What needs to be changed so that it can be useful?
  - For which group of patients will this decision aid not be suitable? → How can we make it more suitable for this group of patients? For which patients will this decision aid be suitable?
- Feasibility (only in alpha-testing round 2)
  - If you are going to use the decision aid in your daily care practice, how would you do that? → how, when and by whom do you want to offer this (or have it offered)?
  - What will this decision aid add to the consultation? → can you explain that further?
  - Will you present this decision aid to your patients? → if no, what makes you not want to use the decision aid? → can we adjust something to make it suitable for you or your patients? And if yes? → what makes you want to use the decision aid?

1. Finally, I have two general questions:

- When the decision aid is ready, will you recommend it to other patients?
- On a scale of 1 to 10, what grade would you give this decision aid?

## D: Acceptability scale

We would like to know what you think of the decision aid 'dose reduction of CML medications'

1. Below you can indicate what you think of each part of the decision aid

Step 1 To reduce dose or not? Poor Fair Good Excellent

Side effects Poor Fair Good Excellent

What does dose reduction involve? Poor Fair Good Excellent

Step 2 What are the differences? Poor Fair Good Excellent

Step 3 What you need to know Poor Fair Good Excellent

Step 4 Considerations Poor Fair Good Excellent

Step 5 What is important to you? Poor Fair Good Excellent

Step 6 Overview Poor Fair Good Excellent

1. Length of the decision aid was (select one answer)

- Too long
- Too short
- Just right

1. The amount of information provided was (select one answer)
   - Too much information
   - Too little information
   - Just right
2. I believe the decision aid (select one answer)
   - Leans towards the choice to reduce the dose
   - Leans towards the choice not to reduce the dose
   - Balanced
3. Would you have found this decision aid useful when making a decision about the dose of your CML medications?
   - Yes
   - No

Comments:

1. Do you find the information provided sufficient to make a choice about dose reduction?
   - Yes
   - No

Comments:

1. Use of difficult words?
   - Many
   - Few
   - None
2. Does the decision aid contain confusing parts?
   - Yes
   - No

If yes, which ones?

1. Navigating through the decision aid is
   - Easy
   - Difficult
   - Neutral
2. Would you recommend this decision aid to CML patients?
   - Yes
   - No

Comments:

1. Rate the decision aid from 1 to 10
2. What did you like about the decision aid?
3. What do you think needs to be improved about the decision aid?

## E: System Usability Scale

With this questionnaire, we would like to assess the usability of the decision aid 'CML medication dose reduction'.

Below are 10 statements regarding the decision aid. For each statement, circle the answer that best fits you, 1 = Strongly disagree; 2 = Disagree; 3 = Neutral; 4 = Agree; 5 = Strongly agree.

|  | Strongly disagree | Disagree | Neutral | Agree | Strongly agree |
| --- | --- | --- | --- | --- | --- |
| I think I would use this decision aid often | 1 | 2 | 3 | 4 | 5 |
| I found the PDA unnecessarily complex | 1 | 2 | 3 | 4 | 5 |
| I found the PDA easy to use | 1 | 2 | 3 | 4 | 5 |
| I think that I would need the support of a technical person to be able to use the PDA | 1 | 2 | 3 | 4 | 5 |
| I found the various functions in this PDA were well integrated | 1 | 2 | 3 | 4 | 5 |
| I thought there was too much inconsistency in this PDA | 1 | 2 | 3 | 4 | 5 |
| I would imagine that most people would learn to use this PDA very quickly | 1 | 2 | 3 | 4 | 5 |
| I found the PDA very cumbersome to use | 1 | 2 | 3 | 4 | 5 |
| I felt confident using the PDA | 1 | 2 | 3 | 4 | 5 |
| I needed to learn a lot of things before I could get going with the PDA | 1 | 2 | 3 | 4 | 5 |

## F: Clarity of each section in the alpha-tested draft version

Supplementary Table 1 - Ratings on the clarity of each section in the alpha-tested draft version of the PDA by both patients and healthcare providers.

|  | | CML patients (n = 17) | | | |  | HCPs (n = 9) | | |
| --- | --- | --- | --- | --- | --- | --- | --- | --- | --- |
| Item | | Poor | Fair | Good | N/A |  | Poor | Fair | Good |
| Introduction  *Situation and possibilities* | Background information | - | 1  (5.9%) | 16  (94.1%) |  |  | - | 1  (11.1%) | 8 (88.9%) |
|  | Dose reduction | - | 1  (5.9%) | 16  (94.1%) |  |  | - | - | 9 (100%) |
|  | Results | - | 1  (5.9%) | 16  (94.1%) |  |  | - | - | 9 (100%) |
|  | Possibilities | - | 1  (5.9%) | 16  (94.1%) |  |  | - | 1  (11.1%) | 8 (88.9%) |
| What are the differences  *Dose reduction or not?* | | - | 1  (5.9%) | 16 (94.1%) |  |  | - | 1  (11.1%) | 8 (88.9%) |
| Important aspects  *Knowledge test* | | - | 2  (22.2%) | 15 (88.2%) |  |  | - | - | 9 (100%) |
| Considerations  *What is important to you* | Considerations | - | 1  (5.9%) | 15 (88.2%) | 1 (5.9%) |  | - | 2  (22.2%) | 7 (77.8%) |
|  | What is important to you | - | 1  (5.9%) | 16 (94.1%) |  |  | - | - | 9 (100%) |
| Summary sheet | | - | 1  (5.9%) | 15 (88.2%) | 1 (5.9%) |  | - | 1  (11.1%) | 8 (88.9%) |

## G: Usability results per scale item and total SUS

Supplementary Table 2 – Usability results per scale item and total SUS. Per scale item the responses of the 5-point Likert scale are presented with their corresponding percentages. ^a^ Mean represents the total point per scale item where “Strongly disagree” = 1 point, “Disagree” = 2 points, etc. n = 16. ^b^ Total SUS score is calculated over the 10 items.

| Scale item | | Mean^a^ (SD) |
| --- | --- | --- |
| 1 | I think I would like to use the PDA frequently | 3.1 (0.9) |
| 2 | I found the PDA unnecessarily complex | 2.4 (1.0) |
| 3 | I found the PDA easy to use | 3.9 (0.9) |
| 4 | I think that I would need the support of a technical person to be able to use the PDA | 2.1 (0.7) |
| 5 | I found the various functions in this PDA were well integrated | 3.7 (0.8) |
| 6 | I thought there was too much inconsistency in this PDA | 2.3 (1.0) |
| 7 | I would imagine that most people would learn to use this PDA very quickly | 3.8 (0.8) |
| 8 | I found the PDA very cumbersome to use | 2.3 (0.9) |
| 9 | I felt confident using the PDA | 3.8 (0.8) |
| 10 | I needed to learn a lot of things before I could get going with the PDA | 2.0 (0.7) |
|  | Total SUS score | 68.1 (17) |
